# Economy in the Generation and Use of Steam

**Published:** 1915-02-13

**Authors:** 


### ECONOMY IN THE GENERATION AND USE OF STEAM.*

### The Condensation of Steam.

It will be understood from what has been said in previous articles that the condensation of the steam, providing that its latent heat can be usefully employed, is the great desideratum for economy in the working of steam plant. The use of exhaust steam for heating various apparatus is the most economical method possible. Some cautions, however, are necessary in the use of steam-heating appliances. One was mentioned in the last article, the deposit of oil from the exhaust steam. It should also be mentioned that when water for hospital purposes is heated, if the water contains some of the salts which go to make it "hard," these salts will tend to deposit on the inside of the tubes through which the water flows. It is, therefore, wise to submit all the water that is heated, for every purpose, to the softening process. The cost of softening the water is comparatively trifling compared to the waste of coal that follows from the deposit of salts mentioned above.

### Trapping the Steam Condensate.

Where steam is used also for heating purposes, care must be taken that the condensed steam, the "condensate," as it is called, is carried away safely to the tank, usually called the "hot well," from which the pumps that feed the boiler take their supply. It is a very simple matter to arrange that all the condensed water from the calorifiers, and from the other apparatus where steam is used for heating, is drained off, and finds its way to the tank provided for it. Also in hospitals it very often happens that long lines of steam-pipe are necessary.

It has been mentioned in a previous article that they should be covered with some thermal insulator to prevent the loss of heat by radiation and convection. No matter how carefully the pipes are covered, however, some condensation of steam takes place; and care must be taken that the "condensate" so formed is trapped at fairly frequent points, and is conveyed away to the hot well. There are a number of "steam traps," as they are called, upon the market. Their forms are very various, but all act upon the same principle. They are connected by a pipe to the lowest points or to low points in the range of steam pipes; and whenever a certain quantity of condensed water collects there a valve arranged for the purpose opens, and allows the water to pass through into a receptacle provided for it, and thence by another pipe to the hot well. The use of steam traps has a double object. It helps to keep up the supply of hot water ready for the boiler feed, and it minimises the possibility of what is known as "water hammer." It sometimes happens, when the above precautions are not taken, that water collects at a low point on the range of steam pipes; sometimes near a valve, sometimes at a bend. The water usually collects when the steam pipes are not in use for a certain time. When steam is allowed to pass through them, after their being out of use, and water having collected, the steam, which is moving at a very high velocity, licks up the water out of the pocket or elbow, and throws it with great violence against any obstruction in its path, such as the end of the pipes, a valve, or any other obstruction. The velocity of the steam is so high that a comparatively small quantity of water propelled may do a great deal of damage.

Care must be taken with all apparatus in which exhaust steam is employed not to set up more back pressure in the steam engine than can be avoided. In the steam engine the steam enters behind the piston, forces it along to the end of the stroke, and on the return stroke, the steam which enters on the other side of the piston has not only to perform whatever work the engine may be engaged upon, but it also has to force out the exhaust steam, the steam which has done its work on the previous stroke in front of it. Anything which prevents the free passage of the exhaust steam adds to the work which the live steam, the steam behind the piston, has to perform, and adds to the amount of coal which has to be burnt in the boiler furnace to do a certain amount of work.

The condenser is employed in power stations, and where steam is not required for other purposes, in order to reduce the back pressure in front of the piston. In the condenser the exhaust steam is condensed to water; and by means of one or two pumps, specially designed for the purpose, the "condensate" is pumped away to the hot well; the air which is also always present with steam, and therefore in the condensate, is also pumped out; and the pressure in front of the piston is reduced to considerably below that of the atmosphere.

Where the exhaust steam from an engine passes into the atmosphere or is utilised for heating, as in hospitals, the back pressure in front of the piston may be as high as 5 lb. per square inch above that of the atmosphere; where a condenser is used the pressure may be reduced to a minus quantity.

* Previous articles on aspects of this subject appeared on January 23, and, in 1914, on May 30, June 13, July 25.

